# Central Infusion of Angiotensin II Type 2 Receptor Agonist Compound 21 Attenuates DOCA/NaCl-Induced Hypertension in Female Rats

**DOI:** 10.1155/2016/3981790

**Published:** 2015-12-13

**Authors:** Shu-Yan Dai, Yu-Ping Zhang, Wei Peng, Ying Shen, Jing-Jing He

**Affiliations:** ^1^Department of Obstetrics and Gynecology, Shengjing Hospital, China Medical University, Shenyang 110004, China; ^2^Department of Pathophysiology, Hebei North University, Zhangjiakou, Hebei 075000, China; ^3^Life Science Research Center, Hebei North University, Zhangjiakou, Hebei 075000, China

## Abstract

The present study investigated whether central activation of angiotensin II type 2 receptor (AT2-R) attenuates deoxycorticosterone acetate (DOCA)/NaCl-induced hypertension in intact and ovariectomized (OVX) female rats and whether female sex hormone status has influence on the effects of AT2-R activation. DOCA/NaCl elicited a greater increase in blood pressure in OVX females than that in intact females. Central infusion of compound 21, a specific AT2-R agonist, abolished DOCA/NaCl pressor effect in intact females, whereas same treatment in OVX females produced an inhibitory effect. Real-time RT-PCR analysis revealed that DOCA/NaCl enhanced the mRNA expression of hypertensive components including AT1-R, ACE-1, and TNF-*α* in the paraventricular nucleus of hypothalamus in both intact and OVX females. However, the mRNA expressions of antihypertensive components such as AT2-R, ACE-2, and IL-10 were increased only in intact females. Central AT2-R agonist reversed the changes in the hypertensive components in all females, while this agonist further upregulated the expression of ACE2 and IL-10 in intact females, but only IL-10 in OVX females. These results indicate that brain AT2-R activation plays an inhibitory role in the development of DOCA/NaCl-induced hypertension in females. This beneficial effect of AT2-R activation involves regulation of renin-angiotensin system and proinflammatory cytokines.

## 1. Introduction

It has been well documented that activation of angiotensin II type 2 receptor (AT2-R) plays a critical role in antagonizing AT1-R overactivity, particularly during pathological conditions [1–3]. Most of early studies looking at short-term or long-term effects of AT2-R revealed that peripheral AT2-R activation did not have an antihypertensive effect but enhanced tissue protection in various hypertensive models [1, 2]. However, recent studies implicated that AT2-R in the central nervous system (CNS) may exert more critical actions on blood pressure (BP) regulation [4]. AT2-R has been shown to reside or be in close proximity to CNS nuclei involved in cardiovascular regulation, including the solitary tract nucleus (NTS), rostral ventrolateral medulla (RVLM), subfornical organ (SFO), and paraventricular nucleus of hypothalamus (PVN) [5, 6]. In particular, within the PVN, the AT2-R-containing neuron fibres and terminals appear to synapse onto preautonomic neuron cell bodies, suggesting that AT2-R can influence sympathetic outflow and BP through these connections [6]. Central blockade of AT2-R in normal male animals attenuates baroreflex control of renal sympathetic nerve activity (RSNA) and heart rate (HR) [7]. In contrast, central activation of AT2-R by intracerebroventricular (icv) infusion of Compound 21 (C21), the first selective nonpeptide AT2-R agonist, or AT2-R overexpression in the RVLM of heart failure animals leads to sympathoinhibition [8, 9], which is accompanied with upregulation of neuronal nitric oxide synthase and downregulation of AT1-R in the several nuclei involved in regulation of BP and sympathetic activity including the PVN [9]. Moreover, central administration of C21 lowered BP and plasma norepinephrine levels in both spontaneous hypertensive (SHR) and WKY male rats. These effects were abolished by coadministration of the AT2-R antagonist PD123319 or the nitric oxide synthase inhibitor N*ω*-nitro-L-arginine methyl ester (L-NAME) hydrochloride [10]. These results indicate that central AT2-R plays an important role in regulation of BP and sympathetic activity in both physiological and pathophysiological states. However, the precise mechanisms underlying the antihypertensive effect of central AT2-R activation remain unclear.

Accumulating evidence indicates that the expression and function of the AT2-R are sexually different. Female sex hormones, especially estrogen, increase the expression of the AT2-R but inhibit the AT1-R expression [11–14]. A series of studies from Denton and colleagues have shown that chronic infusion of a low dose of angiotensin (ANG) II results in an increase in BP in male rats but a decrease in BP in female rats [15]. This depressor effect of ANG II in females is via an AT2-R-mediated and an estrogen-dependent mechanism [15]. Moreover, the AT2-R mediates the normal midgestational decrease in BP and contributes to BP regulation during late gestation [16]. These findings suggest that the BP is differentially regulated by the AT2-R in females as compared with males and support an enhanced role for AT2-R in regulating BP in females. However, these studies focused on the regulating effects of AT2-R in peripheral cardiovascular tissues such as kidney and vasculature. There are few animal studies evaluating the effects of central AT2-R activation on BP regulation in female rats and the influence of female sex hormone status on the effects of central AT2-R activation.

Downregulation of renin-angiotensin system (RAS) hypertensive components, upregulation of RAS antihypertensive components, and anti-inflammation have been shown to be important features of the AT2-R underlying improved outcome in experimental disease models [16–23]. Our previous study has demonstrated that central blockade of AT2-R augments deoxycorticosterone acetate (DOCA)/NaCl-induced pressor effect in females through modulating expression of RAS components and proinflammatory cytokines in the PVN [24]. In the present study, we investigated whether central activation of AT2-R by icv infusion of C21 attenuates DOCA/NaCl-induced hypertension in female rats and whether female sex hormone status has influence on the effects of AT2-R activation. To do so, we employed* in vivo* telemetric recording of BP and real-time quantitative reverse transcription polymerase chain reaction (RT-PCR) assessing mRNA expression of several RAS components and proinflammatory cytokines in the PVN to determine the effects of central activation of AT2-R on the development of DOCA/salt-induced hypertension in intact and ovariectomized (OVX) female rats.

## 2. Methods

### 2.1. Animals

Thirty-six female rats (Wistar, 10–12 wk old) were purchased from Beijing Laboratory Animal Research Center (Beijing, China) and were maintained at an animal facility under barrier-sustained conditions with 12 h light/dark cycle at standard conditions (temperature: 23 ± 2°C, relative humidity: 40%–80%) and free access to standard rat chow* ad libitum*. All animal procedures were reviewed and approved by the China Medical University and the Hebei North University Institutional Animal Care and Use Committee conforming to US National Institutes of Health guidelines.

The female rats were prepared with a lateral ventricular cannula, osmotic minipumps, and DOCA pellet for intracerebroventricular (icv) and subcutaneous drug infusions and with telemetry probes for continuous BP recording, as previously described [24]. Water was also changed to 1% NaCl as the sole drinking fluid. 1% NaCl intakes were measured daily. Thus, the primary study groups (*n* = 6/group) were the following: (1) intact female icv C21 (0.25 *μ*g/h, SPS Alfachem) + 1% NaCl; (2) intact female icv vehicle (V) + DOCA/NaCl; (3) intact female icv C21 + DOCA/NaCl; (4) OVX female icv C21 (0.25 *μ*g/h) + 1% NaCl; (5) OVX female icv vehicle (V) + DOCA/NaCl; and (6) OVX female icv C21 + DOCA/NaCl.

Animals assigned to DOCA treatment were subcutaneously implanted with a DOCA pellet (150 mg/kg, Sigma-Aldrich, USA). After the physiological studies were finished, brains were taken and PVN tissue was collected by micropunching for determining mRNA expression of RAS components and proinflammatory cytokines.

### 2.2. Ovariectomy

Ten days before implantation of telemetry probes, bilateral ovariectomy was performed in female rats anesthetized with pentobarbital sodium (1%, 50 mg/kg). A single 2-3 cm dorsal midline incision was made in the skin and underlying muscles. The ovaries were isolated, tied-off with sterile suture, and removed, and the incisions were closed.

### 2.3. Telemetry Probe Implantation

Under anesthetization with pentobarbital sodium (1%, 50 mg/kg), rats were implanted with telemetry transmitters (TA11-PA40, DSI) through the femoral artery for continuous monitoring of BP and HR.

### 2.4. Chronic Icv Cannula, Osmotic Pump, and DOCA Pellet Implantation

DOCA pellets were made by mixing 30–40 mg DOCA (adjusted by animal body weight) into 1 mL of silicone (Sylgard 184 silicone elastomer base; Dow Corning, Midland, MI). Once the DOCA was homogenously mixed into the silicone, silicone elastomer curing agent (0.2 mL) was added. The DOCA implants were allowed to cure at room temperature for 24 hours and were then stored at 4°C until implantation.

After baseline BP and HR recordings were made, the rats were again anesthetized with pentobarbital sodium, and the icv cannula with an osmotic pump (model 2004, 0.25 *μ*L/h for 4 weeks, ALZET Brain Infusion Kits, Alzet Co.) was implanted into the right lateral ventricle (the coordinates 1.0 mm caudal, 1.5 mm lateral to bregma, and 4.5 mm below the skull surface) for chronic infusion of vehicle or C21 for 4 weeks. At the same time, a pellet of DOCA (150 mg/kg) was implanted subcutaneously in the back and tap water was changed to 1% NaCl.

### 2.5. Real-Time RT-PCR Analysis

At the end of experiments, the animals were euthanized with an overdose of pentobarbital. The brain was removed and quickly frozen on dry ice. Six serial coronal sections (100 *μ*m) were cut through the hypothalamus at the level of the PVN using a cryostat and the PVN region was punched using a blunt 18-gauge needle as previously described [25].

The total RNA was extracted using RNeasy Mini Kit (Qiagen, Valencia, CA, USA) and reverse transcribed into cDNA. mRNA levels for RAS components [AT1-R, AT2-R, angiotensin-converting enzyme- (ACE-) 1, and ACE-2], inflammatory cytokines [tumor necrosis factor- (TNF-)*α* and interleukin- (IL-)10], and GAPDH were analyzed with SYRB Green real-time PCR. The sequences for the primers are summarized in Table 1. Real-time RT-PCR was performed with the ABI prism 7300 Sequence Detection System (Applied Biosystems, Carlsbad, CA). The values were corrected by GAPDH and the final concentration of mRNA was calculated using the formula *x* = 2^−ΔΔCt^, where *x* is fold difference relative to control.

### 2.6. Data Analysis

MAP and HR are presented as mean daily values. Differences for mean arterial pressure (MAP) and HR were calculated for each animal based on the mean of the 3-day baseline subtracted from the mean of the final 5 days of treatment. Two-way ANOVA analysis for the experimental groups was then conducted on the means of calculated differences. After establishing a significant ANOVA,* post hoc* analyses were performed with Tukey multiple comparison tests between pairs of mean changes. The same statistical methods were used to analyze the changes in HR, 1% NaCl intake, and differences in mRNA expression of the RAS components and cytokines in the PVN. All data are expressed as means ± SE. Statistical significance was set at *P* < 0.05.

## 3. Results

### 3.1. Effect of Icv C21 on DOCA/NaCl-Induced Hypertension in Intact Female Rats

**Figure 1:**
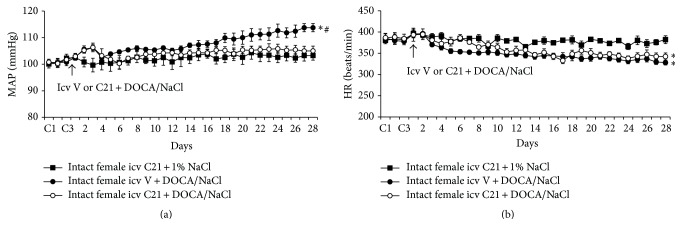
The effect of central infusion of AT2-R agonist, Compound 21 (C21), on DOCA/NaCl-induced increase in blood pressure in intact female rats. Daily mean arterial pressure (MAP) (a) and heart rate (HR) (b) before and during DOCA/NaCl treatment in intact females with or without central infusion of C21. *n* = 6 per group; ^*∗*^ *P* < 0.05 compared to baseline; ^#^ *P* < 0.05 compared to intact females with central infusion of C21.

Icv infusion of C21 plus 1% NaCl had no effects on basal MAP (100.9 ± 1.5 mmHg) and HR (385.6 ± 7.9 beats/min) in intact female rats. DOCA/salt treatment elicited significant increases in MAP in intact females (Δ12.1 ± 1.5 mmHg, *P* < 0.05). Icv infusion of C21 abolished this pressor effect induced by DOCA/NaCl (Δ4.0 ± 1.9 mmHg, *P* < 0.05, Figures 1(a) and 3(a)). In contrast, systemic DOCA infusion resulted in significant, comparable decrease in HR (Figures 1(b) and 3(b), *P* > 0.05) in all groups when compared to intact females receiving C21 plus 1% NaCl.

### 3.2. Effect of Icv C21 on DOCA/NaCl-Induced Hypertension in OVX Female Rats

**Figure 2:**
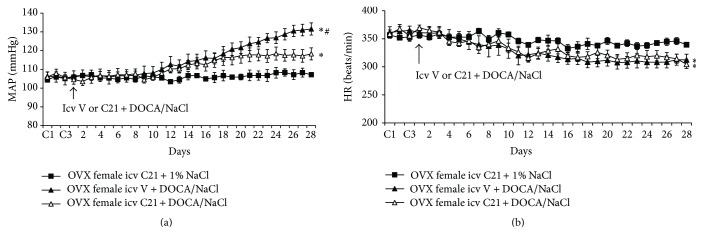
The effect of central infusion of AT2-R agonist, C21, on DOCA/NaCl-induced hypertension in ovariectomized (OVX) rats. Daily mean arterial pressure (MAP) (a) and heart rate (HR) (b) before and during DOCA/NaCl treatment in OVX females with or without central infusion of C21. *n* = 6 per group; ^*∗*^ *P* < 0.05 compared to baseline or central infusion of C21 alone; ^#^ *P* < 0.05 compared to OVX females with central C21 plus DOCA/NaCl.

**Figure 3:**
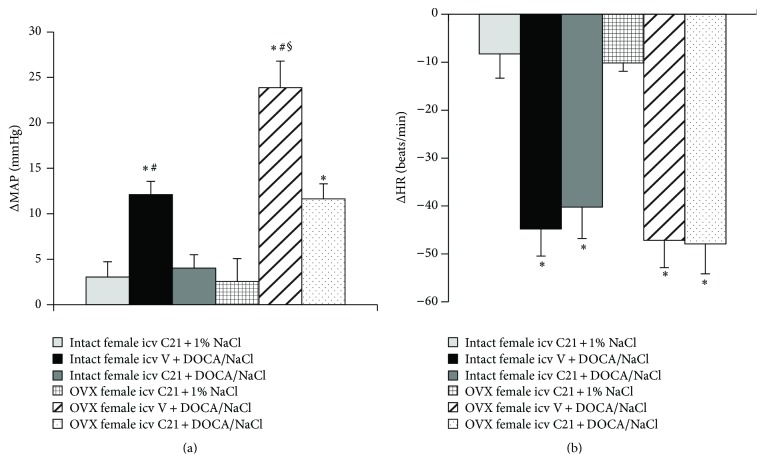
Average changes in MAP (a) and HR (b) produced by DOCA/NaCl treatment in intact and OVX female rats. *n* = 6 per group; ^*∗*^ *P* < 0.05 compared to intact or OVX females with central infusion of C21 alone; ^#^ *P* < 0.05 compared to intact or OVX females with central C21 plus DOCA/NaCl; ^§^ *P* < 0.05 compared to intact females with DOCA/NaCl.

OVX elicited a slight, but significant, increase in baseline MAP (105.8 ± 1.9 mmHg) but decrease in baseline HR (356.8 ± 8.1 beats/min) when compared with intact females (*P* < 0.05). Icv infusion of C21 plus 1% NaCl had no effects on these basal MAP and HR in OVX females. After 4 weeks of DOCA/salt treatment, MAP was remarkably elevated (Δ23.8 ± 2.9 mmHg, *P* < 0.05 versus baseline and intact female group with icv vehicle plus systemic DOCA). Icv infusion of C21 for 4 weeks also attenuated the DOCA/NaCl pressor effect (Δ11.6 ± 1.6 mmHg, *P* < 0.05, Figures 2(a) and 3(a)). Systemic DOCA infusions produced significant, comparable decrease in HR in all groups (Figures 2(b) and 3(b)).

### 3.3. Effect of DOCA Infusion on 1% NaCl Intake

**Figure 4:**
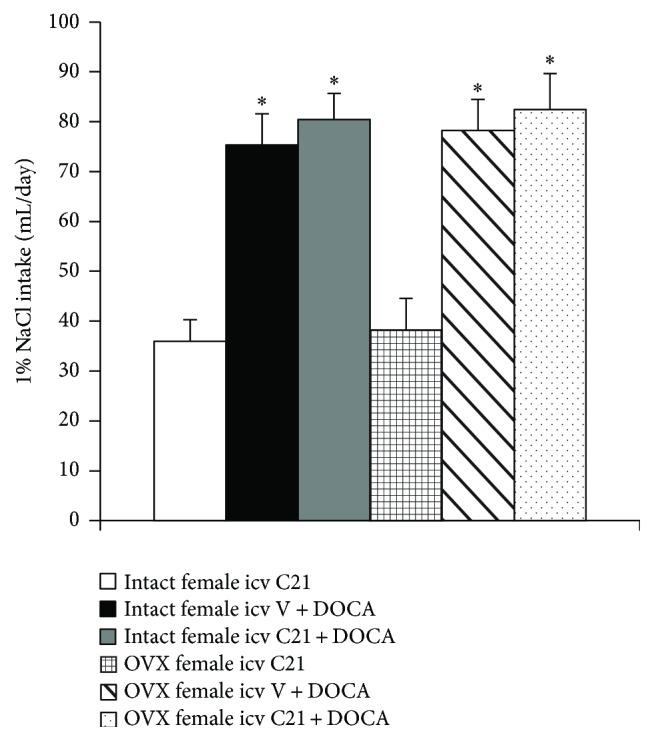
Mean daily 1% NaCl intake during DOCA infusions in female rats treated with central vehicle or C21. *n* = 6 per group; ^*∗*^ *P* < 0.05 compared to intact or OVX females with central infusion of C21 alone.

There was no difference in 1% NaCl intake between intact and OVX female rats when given icv infusions of C21 alone. Systemic infusion of DOCA produced a significant, but comparable, increase in 1% NaCl intake in all groups of rats (Figure 4).

### 3.4. Effect of Icv C21 on mRNA Expression of RAS Components and Inflammatory Cytokines in the PVN

**Figure 5:**
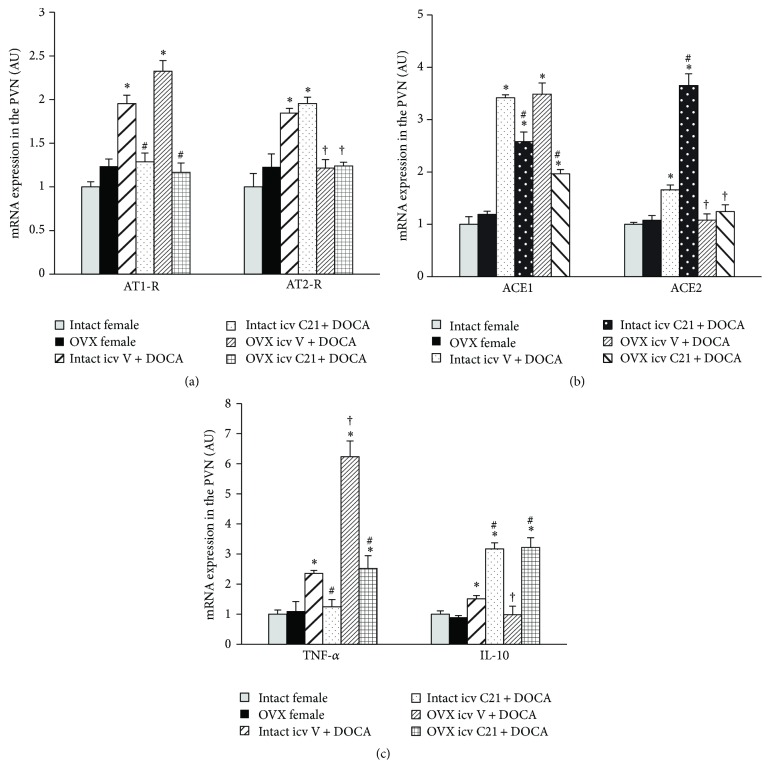
mRNA levels for renin-angiotensin system components (AT1-R and AT2-R (a); ACE1 and ACE2 (b)) and inflammatory cytokines (TNF-*α* and IL-10 (c)) in the PVN in each group of females with or without central infusion of C21 during DOCA/NaCl treatment. Values are mean ± SEM and expressed as a fold change relative to corresponding control (intact females). *n* = 6 per group; ^*∗*^ *P* < 0.05 compared to control intact females; ^#^ *P* < 0.05 compared to intact and OVX females with central vehicle plus systemic DOCA/NaCl. ^†^ *P* < 0.05 compared to intact females with central vehicle plus systemic DOCA/NaCl.

In PVN tissue collected from intact females, DOCA/NaCl upregulated the mRNA expression of both hypertensive components (AT1-R, ACE1, and TNF-*α*) and antihypertensive components (AT2-R, ACE2, and IL-10) within RAS and inflammatory cytokines when compared with controls (*P* < 0.05). Central infusion of C21 reversed the changes in mRNA expression of AT1-R, ACE1, and TNF-*α*. In contrast, the mRNA expressions of ACE2 and IL-10 were further increased (*P* < 0.05, Figure 5) while the mRNA expression of AT2-R remained higher.

Ovariectomy alone had no effect on the mRNA expression of RAS components and inflammatory cytokines in the PVN. In these OVX females, DOCA infusion resulted in a significant increase in the mRNA expression of AT1-R, ACE1, and TNF-*α* in the PVN (*P* < 0.05, Figure 5) while the expressions of AT2-R, ACE2, and IL-10 were not altered. Central infusion of C21 reduced these increased expressions of AT1-R, ACE1, and TNF-*α* while IL-10 expression was elevated during DOCA infusion (*P* < 0.05, Figure 5). The mRNA expression of ACE2 and AT2-R remained unchanged.

## 4. Discussion

The major findings of the present study are as follows: (1) central activation of AT2-R abolished DOCA/NaCl pressor effect in intact females, whereas same treatment in OVX females produced an inhibitory effect; (2) DOCA/NaCl treatment resulted in a greater increase in BP in OVX females, which was accompanied with increased mRNA expression of AT1-R, ACE1, and TNF-*α*, but with no altered expression of AT2-R, ACE2, and IL-10 in the PVN when compared to intact females; these changes in gene expression may be responsible for the augmentation of pressor effects induced by DOCA/NaCl in OVX females; and (3) central infusion of AT2-R agonist C21 reversed the changes in the hypertensive components in all females, while this agonist further upregulated the expression of ACE2 and IL-10 in intact females, but only IL-10 in OVX females, suggesting different mechanism involving the AT2-R regulation of antihypertensive components of the RAS between intact and OVX females. These results indicate that activation of AT2-R in the CNS plays an inhibitory role in the development of DOCA/salt-induced hypertension in females and that this antihypertensive effect involves regulation of the RAS and proinflammatory cytokines.

Whether AT2-R activation alone is sufficient to lower BP has been debated. Some studies showed the antihypertensive effect of systemic AT2-R activation only in the presence of AT1-R blockers or ACE inhibitors [1, 2], whereas others demonstrated a direct depressor effect of systemic AT2-R activation [20, 23]. Hussain and colleagues reported that oral administration of C21 prevents salt-sensitive hypertension in obese Zucker rats and that this protective effect of C21 is associated with activation of renal AT2-R and improvement of renal function [23]. However, Hilliard et al. did not observe an antihypertensive effect of AT2-R activation in male SHRs [26]. Recent studies revealed that activation of AT2-R locally within the brain, without manipulating AT1-R or ACE activity, results in a BP lowering effect. Gao and colleagues reported that there is robust expression of AT2-R in the brain and that central overexpression or activation of AT2-R by C21 reduces sympathetic outflow and BP in male rats [5, 6, 8, 9]. Brouwers and colleagues also demonstrated central administration of C21 lowered BP and plasma norepinephrine levels in both spontaneous hypertensive and WKY male rats [10]. These results suggest that central AT2-R negatively regulates neuronal function and cardiovascular activity, thereby reducing BP.

In human and animal hypertensive models, sex differences in the regulation of BP have been established, possibly through differences in the function of the RAS and in response to stimulation and inhibition of RAS between males and females. Accumulating evidence shows that the expression and function of the AT2-R are enhanced in females [11–13], suggesting that the activation of AT2-R in females may play a more potentiated role. In the female rats, the elevated BP induced by systemic ANG II infusion was markedly reduced when C21 was concomitantly infused intrarenally [27]. Hilliard et al. reported that AT2-R stimulation increases renal function in female, but not in male SHR rats [26]. Our previous studies also showed that central blockade of AT2-R augmented increase in the BP induced by DOCA/NaCl in female while the same treatment had no effect in male rats, suggesting that central AT2-R in females plays an enhanced protective role [24]. The present study extends our previous work by showing that icv infusion of AT2-R agonist abolished and inhibited DOCA/NaCl pressor effect in intact and OVX females, respectively. This study provides direct evidence that activation of central AT2-R is also sufficient to reduce the increase in BP induced by DOCA/NaCl in females.

Within the RAS, ACE1/ANG II/AT1-R has been considered as a hypertensive axis while ACE2/ANG-(1–7)/Mas-R and ANG II/AT2-R have been viewed as an antihypertensive axis [28]. Likewise, the proinflammatory cytokines such as TNF-*α* are involved in the development and the maintenance of hypertension. In contrast, IL-10 plays a protective role against hypertension [29–31]. It has been established that female sex hormones play a critical role in regulating the expression of the RAS and cytokines, with downregulation of hypertensive components including AT1-R, ACE1, and TNF-*α* and upregulation of antihypertensive components including AT2-R, ACE2, and IL-10 [11, 16, 17]. In the present study, we found that DOCA/NaCl treatment upregulated expression of AT1-R, ACE1, and TNF-*α* in the PVN in both intact and OVX females, while the mRNA expression of AT2-R, ACE2, and IL-10 was upregulated only in intact females, but not in OVX females. Given the counterregulatory effects of the AT2-R, ACE2, and IL-10 on AT1-R, ACE1, and TNF-*α* overactivity, these results implicate that increased expression of AT2-R, ACE2, and IL-10 may play a protective role in the development of hypertension in intact females. The results also suggest that the female sex hormone status makes a different way in the RAS and cytokines where intact female and OVX female respond to physiological and pathophysiological stimulations, and female sex hormones shift the balance of the RAS and proinflammatory cytokines to favor the antihypertensive elements.

It has been shown that AT2-R is expressed to a greater extent in the kidney and vasculature of female rats and mice when compared to respective males [32]. In the CNS, Rodriguez-Perez and colleagues reported that the basal mRNA and protein expressions of AT2-R in the substantia nigra were higher in females with high level of estrogen during estrous cycle than in males. Estrogen replacement reversed ovariectomy-induced decrease in AT2-R expression in same nucleus [12, 13]. Our previous and current study showed only a slight, but not significant increase in AT2-R expression in the PVN of female rats when compared to male rats [24]. However, after DOCA/NaCl treatment, AT2-R expression in the PVN was significantly increased in intact females, but not in males and OVX females. These results suggest that female sex hormone status has an influence on the expression of AT2-R, especially under the pathophysiological condition, and that AT2-R plays a role in opposing the pressor actions induced by hypertensive component activation in females via an estrogen-dependent mechanism.

Accumulating evidence demonstrates that long-term AT2-R activation increases kidney ACE2 expression and activity, the Mas receptor (MasR), and its ligand ANG-(1–7) as well as IL-10 level but attenuates AT1-R and TNF-*α* expression in obese Zucker rats. Conversely, blockade of AT2-R by PD123,319 reversed the changes of these genes or agents [18–20, 23]. In* in vitro* studies, AT2-R stimulation exerts an anti-inflammatory action in renal epithelial cells, THP-1 macrophages, and human monocytic cells via reduced TNF-*α* and enhanced IL-10 production. IL-10 was critical for the anti-inflammatory effects of AT2-R stimulation because the IL-10-neutralizing antibody dose-dependently abolished the AT2R-mediated decrease in TNF-*α* level [19, 21, 22]. In the present study, we found that, in both intact and OVX female rats, central activation of AT2 not only downregulated expression of AT1-R, ACE1, and TNF-*α*, but also upregulated expression of IL-10 in the PVN. The changes in these gene expressions may be responsible for the AT2-R attenuation of DOCA/NaCl-induced hypertension in female rats, independent of female sex hormone status. Moreover, we found that central AT2-R activation further upregulated the expression of ACE2 while the AT2-R was kept higher in intact females, but not in OVX females, suggesting that female sex hormones are involved in the AT2-R regulation of antihypertensive components of the RAS and that the protective role of ACE2 was lost in the OVX females. These may be the explanations for the blocking effect of AT2-R activation on DOCA/NaCl-induced increase in BP in intact females and for only attenuating effect of AT2-R activation in the OVX females.

In addition, although OVX female rats showed a greater increase in BP response to DOCA than intact female rats, and central AT2-R activation altered the BP responses to DOCA infusion in all female rats in the present studies, saline intakes and decreases in HR in all groups were similar regardless of female sex hormone status or treatment condition. Thus, the differences in DOCA-induced hypertension between intact and OVX females and the effects of central AT2-R activation on DOCA-induced hypertension in the present study are unlikely to be due to saline intakes and HR changes, which is consistent with the previous studies [33].

It should be noted that there are several limitations in the present study. The present study determined the mRNA expression in a single cardiovascular autonomic nucleus, the PVN, after central C21 and DOCA/NaCl treatment. However, the protective role of central AT2-R activation in the development of DOCA-induced hypertension cannot be attributed solely to the changes in gene expression in the PVN. The central AT2-R activation-induced alterations of gene expression in other cardiovascular regulatory centers such as the nucleus of solitary tract, a nucleus with robust expression of AT2-R [6], may also contribute to the protective role of AT2-R activation in DOCA-induced hypertension. In addition, the possibility cannot be ruled out that the changes in BP produced by DOCA/NaCl and C21 treatment have an influence on the expression of the RAS components and proinflammatory cytokines in the PVN, and the studies on the time sequence of changes in mRNA expression in the PVN relative to the pressor response should be performed in the future.

Taken together, female sex hormone status has an influence on mRNA expression of central AT2-R and its regulation of antihypertensive components of the RAS such as ACE2 expression. Nonetheless, central activation of AT2-R inhibited hypertensive components and enhanced antihypertensive components in the brain nucleus involved in regulation of cardiovascular function, thereby decreasing the BP induced by DOCA/NaCl in both intact and OVX females. The present study extends the previous studies focusing on the effect of AT2-R in peripheral tissues and provides a new central mechanism responsible for the antihypertensive effect of AT2-R activation in females.

## Figures and Tables

**Table 1:** Sequences for primers.

Gene	Gene ID	Primers	Sequences
AT1-R	NM_030985	Forward	5′-CTCAAGCCTGTCTACGAAAATGAG-3′
		Reverse	5′-GTGAATGGTCCTTTGGTCGT-3′

AT2-R	NM_012494	Forward	5′-TGCTGTTGTGTTGGCATTCA-3′
		Reverse	5′-ATCCAAGAAGGTCAGAACATGGA-3′

ACE-1	NM_012544	Forward	5′-TTTGCTACACAAATGGCACTTGT-3′
		Reverse	5′-CGGGACGTGGCCATTATATT-3′

ACE-2	NM_001012006	Forward	5′-TTGAACCAGGATTGGACGAAA-3′
		Reverse	5′-GCCCAGAGCCTACGATTGTAGT-3′

TNF-*α*	NM_013693	Forward	5′-GCATGATCCGCGACGTGGAA-3′
		Reverse	5′-AGATCCATGCCGTTGGCCAG-3′

IL-10	NM_012854	Forward	5′-GTTGCCAAGCCTTGTCAGAAA-3′
		Reverse	5′-TTTCTGGGCCATGGTTCTCT-3′

GAPDH	NM_017008	Forward	5′-GCCAAAAGGGTCATCATCTC-3′
		Reverse	5′-GGCCATCCACAGTCTTCT-3′

AT-R, angiotensin receptor; ACE, angiotensin converting enzyme; TNF-*α*, tumor necrosis factor-*α*; IL-10, interleukin-10.

## References

[1] Steckelings U. M., Paulis L., Namsolleck P., Unger T. (2012). AT2 receptor agonists: hypertension and beyond. *Current Opinion in Nephrology and Hypertension*.

[2] Sumners C., de Kloet A. D., Krause E. G., Unger T., Steckelings U. M. (2015). Angiotensin type 2 receptors: blood pressure regulation and end organ damage. *Current Opinion in Pharmacology*.

[3] Matavelli L. C., Siragy H. M. (2015). AT2 receptor activities and pathophysiological implications. *Journal of Cardiovascular Pharmacology*.

[4] Gao L., Zucker I. H. (2011). AT2 receptor signaling and sympathetic regulation. *Current Opinion in Pharmacology*.

[5] Gao J., Chao J., Parbhu K.-J. K. (2012). Ontogeny of angiotensin type 2 and type 1 receptor expression in mice. *Journal of the Renin-Angiotensin-Aldosterone System*.

[6] de Kloet A. D., Wang L., Ludin J. A. (2014). Reporter mouse strain provides a novel look at angiotensin type-2 receptor distribution in the central nervous system. *Brain Structure and Function*.

[7] Abdulla M. H., Johns E. J. (2014). Nitric oxide impacts on angiotensin AT2 receptor modulation of high-pressure baroreflex control of renal sympathetic nerve activity in anaesthetized rats. *Acta Physiologica*.

[8] Gao L., Wang W., Wang W., Li H., Sumners C., Zucker I. H. (2008). Effects of angiotensin type 2 receptor overexpression in the rostral ventrolateral medulla on blood pressure and urine excretion in normal rats. *Hypertension*.

[9] Gao J., Zucker I. H., Gao L. (2014). Activation of central Angiotensin type 2 receptors by compound 21 improves arterial baroreflex sensitivity in rats with heart failure. *American Journal of Hypertension*.

[10] Brouwers S., Smolders I., Wainford R., Dupont A. (2015). Hypotensive and sympathoinhibitory responses to selective central AT_2_ receptor stimulation in spontaneously hypertensive rats. *Clinical Science*.

[11] Denton K. M., Hilliard L. M., Tare M. (2013). Sex-related differences in hypertension: seek and ye shall find. *Hypertension*.

[12] Rodriguez-Perez A. I., Valenzuela R., Villar-Cheda B., Guerra M. J., Lanciego J. L., Labandeira-Garcia J. L. (2010). Estrogen and angiotensin interaction in the substantia nigra. Relevance to postmenopausal Parkinson's disease. *Experimental Neurology*.

[13] Rodriguez-Perez A. I., Valenzuela R., Villar-Cheda B., Guerra M. J., Labandeira-Garcia J. L. (2012). Dopaminergic neuroprotection of hormonal replacement therapy in young and aged menopausal rats: role of the brain angiotensin system. *Brain*.

[14] Kisley L. R., Sakai R. R., Fluharty S. J. (1999). Estrogen decreases hypothalamic angiotensin II AT1 receptor binding and mRNA in the female rat. *Brain Research*.

[15] Sampson A. K., Hilliard L. M., Moritz K. M. (2012). The arterial depressor response to chronic low-dose angiotensin II infusion in female rats is estrogen dependent. *The American Journal of Physiology—Regulatory Integrative and Comparative Physiology*.

[16] Mirabito K. M., Hilliard L. M., Wei Z. (2014). Role of inflammation and the angiotensin type 2 receptor in the regulation of arterial pressure during pregnancy in mice. *Hypertension*.

[17] McCarthy C. A., Widdop R. E., Denton K. M., Jones E. S. (2013). Update on the angiotensin AT_2_ receptor. *Current Hypertension Reports*.

[18] Ali Q., Wu Y., Hussain T. (2013). Chronic AT2 receptor activation increases renal ACE2 activity, attenuates AT1 receptor function and blood pressure in obese Zucker rats. *Kidney International*.

[19] Dhande I., Ali Q., Hussain T. (2013). Proximal tubule angiotensin AT2 receptors mediate an anti-inflammatory response via interleukin-10: role in renoprotection in obese rats. *Hypertension*.

[20] Sabuhi R., Ali Q., Asghar M., Al-Zamily N. R. H., Hussain T. (2011). Role of the angiotensin II AT2 receptor in inflammation and oxidative stress: opposing effects in lean and obese Zucker rats. *American Journal of Physiology—Renal Physiology*.

[21] Dhande I., Ma W., Hussain T. (2015). Angiotensin AT_2_ receptor stimulation is anti-inflammatory in lipopolysaccharide-activated THP-1 macrophages via increased interleukin-10 production. *Hypertension Research*.

[22] Menk M., Graw J. A., von Haefen C. (2015). Stimulation of the angiotensin II AT2 receptor is anti-inflammatory in human lipopolysaccharide-activated monocytic cells. *Inflammation*.

[23] Ali Q., Patel S., Hussain T. (2015). Angiotensin AT2 receptor agonist prevents salt-sensitive hypertension in obese Zucker rats. *The American Journal of Physiology—Renal Physiology*.

[24] Dai S. Y., Peng W., Zhang Y. P., Li J. D., Shen Y., Sun X. F. (2015). Brain endogenous angiotensin II receptor type 2 (AT2-R) protects against DOCA/salt-induced hypertension in female rats. *Journal of Neuroinflammation*.

[25] Zheng H., Sharma N. M., Liu X., Patel K. P. (2012). Exercise training normalizes enhanced sympathetic activation from the paraventricular nucleus in chronic heart failure: role of angiotensin II. *American Journal of Physiology—Regulatory Integrative and Comparative Physiology*.

[26] Hilliard L. M., Chow C. L. E., Mirabito K. M. (2014). Angiotensin type 2 receptor stimulation increases renal function in female, but not male, spontaneously hypertensive rats. *Hypertension*.

[27] Kemp B. A., Howell N. L., Gildea J. J., Keller S. R., Padia S. H., Carey R. M. (2014). AT2 receptor activation induces natriuresis and lowers blood pressure. *Circulation Research*.

[28] Xu P., Sriramula S., Lazartigues E. (2011). ACE2/ANG-(1–7)/Mas pathway in the brain: the axis of good. *American Journal of Physiology—Regulatory, Integrative and Comparative Physiology*.

[29] Song X.-A., Jia L.-L., Cui W. (2014). Inhibition of TNF-*α* in hypothalamic paraventricular nucleus attenuates hypertension and cardiac hypertrophy by inhibiting neurohormonal excitation in spontaneously hypertensive rats. *Toxicology and Applied Pharmacology*.

[30] Sriramula S., Cardinale J. P., Francis J. (2013). Inhibition of TNF in the brain reverses alterations in RAS components and attenuates angiotensin II-induced hypertension. *PLoS ONE*.

[31] Winklewski P. J., Radkowski M., Wszedybyl-Winklewska M., Demkow U. (2015). Brain inflammation and hypertension: the chicken or the egg?. *Journal of Neuroinflammation*.

[32] Hilliard L. M., Sampson A. K., Brown R. D., Denton K. M. (2013). The ‘His and Hers’ of the renin-angiotensin system. *Current Hypertension Reports*.

[33] Xue B., Badaue-Passos D., Guo F., Gomez-Sanchez C. E., Hay M., Johnson A. K. (2009). Sex differences and central protective effect of 17*β*-estradiol in the development of aldosterone/NaCl-induced hypertension. *American Journal of Physiology: Heart and Circulatory Physiology*.

